# Appendiceal Bulge on Routine Colonoscopy: Not All Disease Is Luminal

**DOI:** 10.7759/cureus.35466

**Published:** 2023-02-25

**Authors:** Sobaan Taj, Usman Ali Akbar, Shawn Philip, Brett Miller, ZakaUl Haq, Harshavardhan Sanekommu, Mohammad A Hossain, Michael Sciarra

**Affiliations:** 1 Internal Medicine, Hackensack Meridian Jersey Shore University Medical Center, Neptune City, USA; 2 Gastroenterology, North Shore University Hospital, Manhasset, USA; 3 Gastroenterology, Hackensack Meridian Palisades Medical Center, North Bergen, USA; 4 Internal Medicine, Raritan Bay Medical Center, Perth Amboy, USA; 5 Internal Medicine, Hackensack Meridian School of Medicine, Nutley, USA

**Keywords:** appendiceal tumors, appendiceal, appendiceal masses, appendiceal mucinous neoplasm, appendiceal mucocele, appendiceal bulge

## Abstract

*Appendiceal mucocele* is an extremely rare pathology accounting for 0.3-0.7% of all appendiceal pathology. It is characterized by appendiceal lumen dilatation by mucinous secretion collection. Though abdominal imaging and tissue Biopsy aids in diagnosis, suspicion should arise when a slight bulge or protrusion is seen on colonoscopy. We present a case of incidental appendiceal bulge found on a routine colonoscopy to evaluate abdominal pain that led to prompt diagnosis and management of appendiceal mucocele.

## Introduction

Rokitansky first described appendiceal mucoceles as localized or diffuse dilatation of the appendiceal lumen by abnormal accumulation of the mucus [1]. They represent 0.3-0.7% of all appendiceal pathology, and an estimated 3500 cases are annually in the United States. It is more common in females and people older than 50 [2]. It is vital to diagnose this rare pathology as it is usually missed and often discovered incidentally in patients requiring endoscopic evaluation for unrelated complaints. We present a case of incidental appendiceal bulge found on a routine colonoscopy to evaluate abdominal pain that eventually led to a diagnosis of an appendiceal mucocele.

## Case presentation

**Figure 1:**
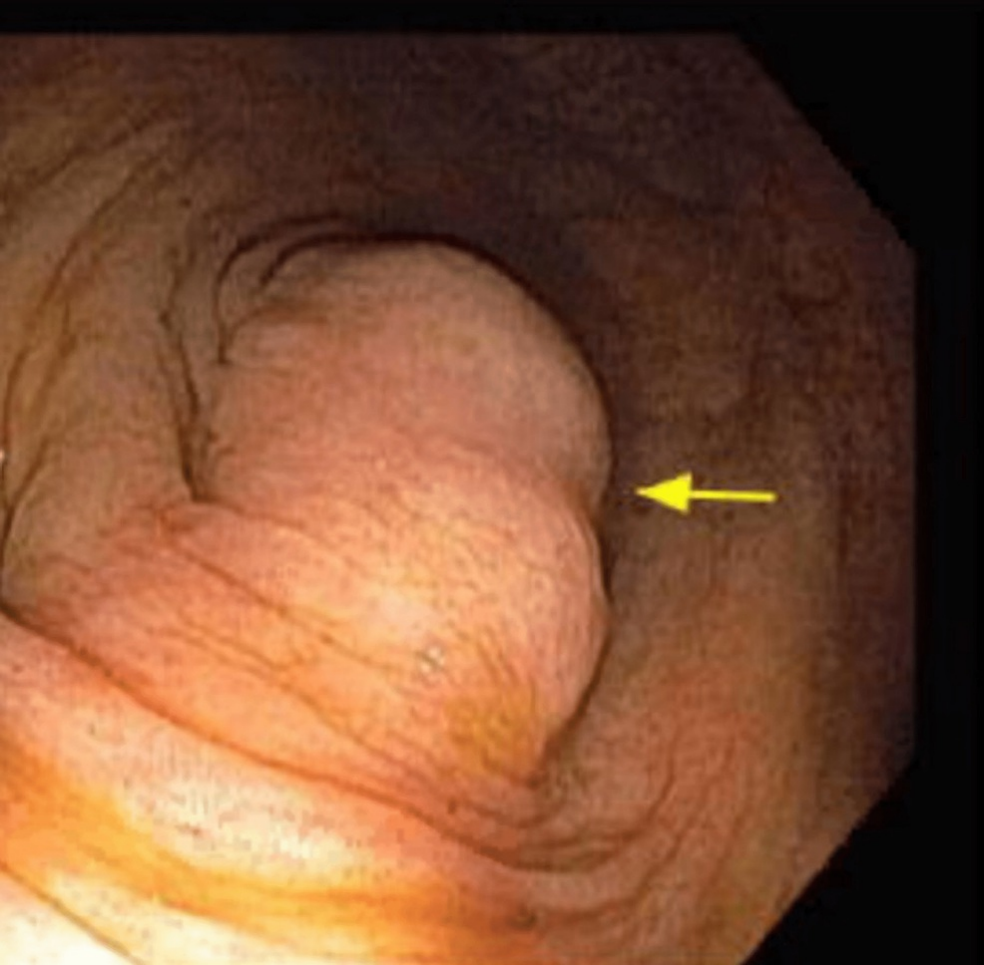
Colonoscopy image from Cecum demonstrating an arrow pointing towards the bulge covering the Appendiceal orifice.

**Figure 2:**
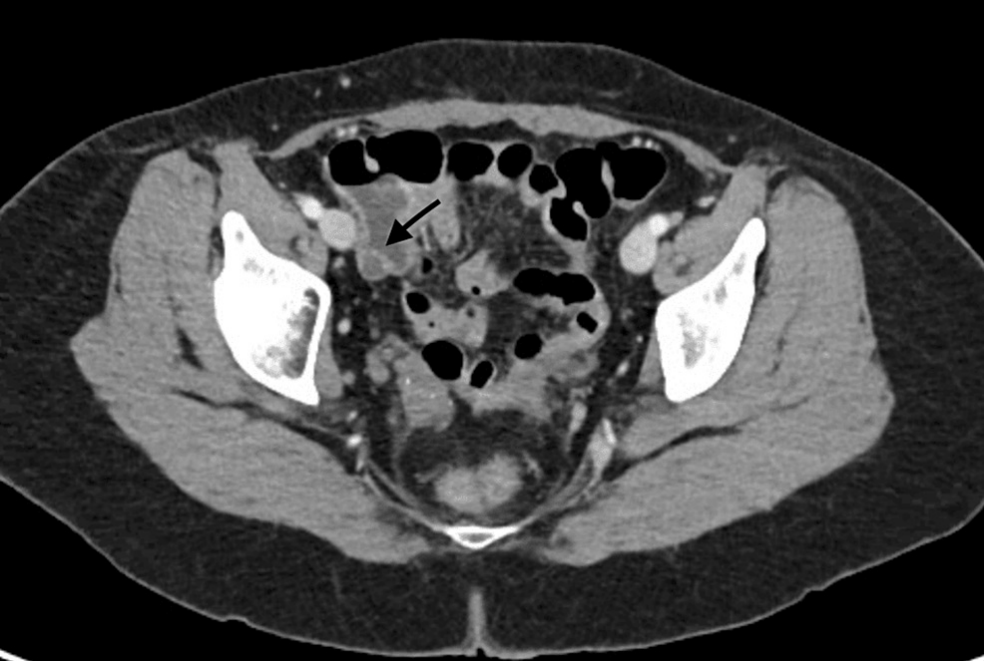
CT scan of the abdomen and pelvis without contrast showing an arrow pointing towards a mass in the Appendiceal region.

A 55-year-old Caucasian female with a past medical history of gastric polyps and a family history of colon cancer presented to the hospital for a routine colonoscopy. On pre-procedure evaluation patient also reported chronic intermittent mild diffuse abdominal pain, non-radiating with no alleviating or aggravating factors associated with on and off non-bloody diarrhea for the last two years. The patient denied anorexia or weight loss. The patient underwent a routine colonoscopy, which showed a bulge right next to the appendiceal orifice (Figure 1). A computer tomography (CT) scan of the abdomen and pelvis was completed to evaluate the appendiceal abnormality further. Imaging revealed a rounded central low-density mass measuring 6.0 × 1.3 × 1.7 cm. It was primarily confined to the proximal appendix, with concern for possible neoplasm (figure 2).

**Figure 3:**
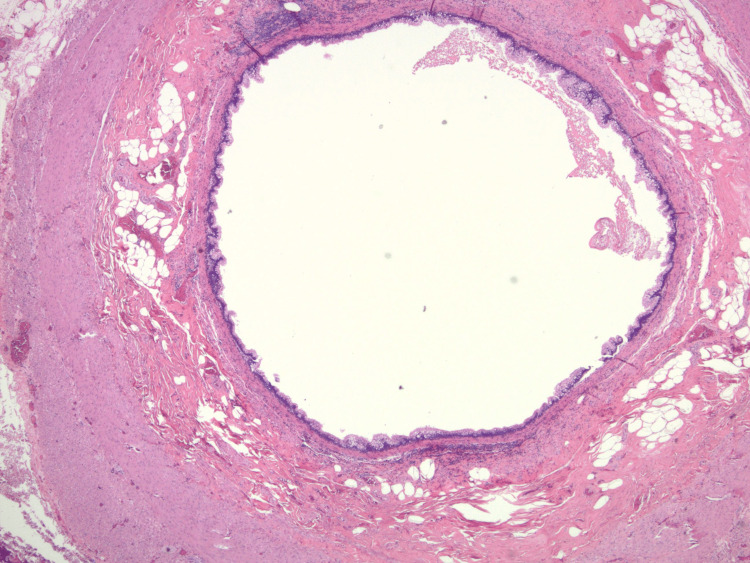
Biopsy image demonstrating dilated Appendiceal lumen filled with mucin and lined by mucinous epithelium.

Due to worsening symptomatology, she presented again to the hospital with right lower quadrant pain. She underwent an emergent hand-assisted laparoscopic appendectomy with resection of the base of the cecum. The appendiceal specimen taken during the procedure was positive for low-grade mucinous neoplasm (Figure 3). The surgery was successful, and the patient had an excellent postoperative outcome with a safe discharge. 

## Discussion

Mucinous appendiceal neoplasms (MAN) are rare entities in the domain of appendiceal tumors, characterized by neoplastic epithelial cells containing abundant cytoplasmic mucin secreted in the lumen of the appendix [3,4]. These tumors have a high tendency for peritoneal metastasis, and their molecular pathogenesis can involve KRAS gene mutations. Recent data suggests that the KRAS mutation may predict worse progression-free survival but not overall survival [5]. As many patients have metastatic disease at diagnosis, it is essential to actively look for appendiceal abnormalities, such as bulges or protrusions, during a routine colonoscopy. Although 23-50% of the patients are asymptomatic at presentation, MAN can present as pain in the lower quadrant of the abdomen, a palpable abdominal mass, nausea, vomiting, weight loss, or gastrointestinal bleeding. These tumors can be found incidentally on routine colonoscopy, but a CT scan is the gold standard for diagnosing these tumors [6]. After the diagnosis, surgical removal of the tumor is paramount. Laparoscopic appendectomy for mucocele is performed, but laparoscopic colectomy is becoming the mainstream management option [7]. A perforated mucocele, enlarged mesenteric lymph node, or positive cytology can suggest a malignant mucocele for which a right hemicolectomy is recommended [8]. The risk of colonic adenocarcinoma is six times greater in these patients than in the general population; thus, surveillance colonoscopy may also be indicated [3]. The low-grade epithelial dysplasia of appendiceal mucinous neoplasms (LAMNs) is characterized by mildly enlarged hyperchromatic nuclei with minimal mitotic activity. The expansile growth of the lamina may push on the appendiceal wall leading to rupture [9].

In cases where radical appendectomy is not viable, Right hemicolectomy is the standard care procedure. However, open surgery is a better option for large and giant tumors due to the risk of iatrogenic rupture and mucin spread in the peritoneal cavity [7]. In our case, the laparoscopic approach was undertaken to perform a cecectomy with hand-assisted appendectomy.

Patita et al. (2020) presented a 42-year-old male with persistent abdominal pain and constipation for one year, for which he underwent a colonoscopy that showed appendiceal mucocele. Biopsy taken during the colonoscopy resulted in drainage of the mucinous fluid [10]. Like our case, persistent abdominal pain led to further luminal evaluation with colonoscopy and, ultimately, a diagnosis. Another case by Grewal et al. (2021) presented a 62-year-old male who underwent a surveillance colonoscopy that incidentally found an appendiceal mucocele on luminal evaluation by appreciating a submucosal non-hemorrhaging 1cm benign appearing lesion [11].

## Conclusions

Appendiceal mucocele is a rare entity that can be missed during a routine colonoscopy. We emphasize that tissue changes and a protuberance or bulge around the appendiceal orifice on colonoscopy should prompt further evaluation. This may allow treatment to be instituted before metastasis if an appendiceal mucocele is found. The laparoscopic approach is the standard of care for patients with subsequent removal of appendiceal tumors.

Recognition of a mucocele bulge during a colonoscopy is critical because it may pave the way for a correct diagnosis and guide further care. Cases like ours emphasize the utility of colonoscopy to aid in examining the root of persistent gastrointestinal complaints and diagnosing underlying pathology beyond the visible lumen on colonoscopy.

## References

[1] Dachman AH, Lichtenstein JE, Friedman AC (1985). Mucocele of the appendix and pseudomyxoma peritonei. Am J Roentgenol.

[2] Choudry HA, Pai RK (2018). Management of mucinous appendiceal tumors. Ann Surg Oncol.

[3] Lohsiriwat V, Vongjirad A, Lohsiriwat D (2009). Incidence of synchronous appendiceal neoplasm in patients with colorectal cancer and its clinical significance. World J Surg Oncol.

[4] Persaud T, Swan N, Torreggiani WC (2007). Giant mucinous cystadenoma of the appendix. Radiographics.

[5] Nishikawa G, Sekine S, Ogawa R (2013). Frequent GNAS mutations in low-grade appendiceal mucinous neoplasms. Br J Cancer.

[6] Shaib WL, Assi R, Shamseddine A (2017). Appendiceal mucinous neoplasms: diagnosis and management. Oncologist.

[7] Young S, Sueda SK, Hotta M, Sung ML, OʼConnor VV, Leung AM (2020). Surgical management of appendiceal mucinous neoplasm: is appendectomy sufficient?. J Surg Oncol.

[8] Vyas J, Badgurjar M, Saxena P, Parihar S, Thakor P (2021). Prudent planning in management of mucocele of appendix. Int J Surg Case Rep.

[9] Ning S, Yang Y, Wang C, Luo F (2019). Pseudomyxoma peritonei induced by low-grade appendiceal mucinous neoplasm accompanied by rectal cancer: a case report and literature review. BMC Surg.

[10] Patita M, Nunes G, Fernandes V (2020). An appendiceal mucocele as an unexpected finding from colonoscopy. Clin Gastroenterol Hepatol.

[11] Grewal JS, Berger E, Garner J, Mayer SL, Beaty JS (2021). Surveillance colonoscopy revealing asymptomatic low-grade appendiceal mucinous neoplasm. Cureus.

